# Associations Between Short-Video Platform Use and Health Across Health Distribution and Usage Behaviors in China: Cross-Sectional Questionnaire Study

**DOI:** 10.2196/86526

**Published:** 2026-03-12

**Authors:** Yangyang Pan, Kangkang Zhang, Yilin Wei, Yangzhen Huang, Chengxu Long, Chenxin Yang, Shangfeng Tang

**Affiliations:** 1 School of Medicine and Health Management, Huazhong University of Science and Technology, No. 13, Aviation Road, Qiaokou District, Wuhan, China, 86 3349895639; 2 Research Center for Rural Health Service, Key Research Institute of Humanities & Social Sciences of Hubei Provincial Department of Education, Wuhan, China

**Keywords:** short-video platform, self-rated health, quantile regression, health disparities, socioeconomic status

## Abstract

**Background:**

Short-video platforms, characterized by algorithmic curation and passive consumption, have emerged as dominant components of digital life. However, the associations between short-video platform use and health across different groups and usage behaviors remain understudied.

**Objective:**

This study investigates associations between short-video platform use and health, examining whether these relationships vary across health status, usage behaviors, and socioeconomic status.

**Methods:**

A cross-sectional study was conducted using multistage stratified sampling across eastern, central, and western China from July to September 2024. The inclusion criteria were age 18 years or older, ability to communicate effectively, and no cognitive disorders or mental disturbance. Of 7725 participants enrolled, 46.96% (n=3628) were male, and the average age was 65.49 (SD 8.39) years. The data were collected via face-to-face interviews using a structured questionnaire. Self-rated health and relative health deprivation (Kakwani index) were used to measure health. Quantile regression explored associations between whether using short-video platform and health varies across the health distribution, while linear regression examined associations of years, frequency, daily duration, and purpose diversity of short-video platform use with health. Moderating effect analysis explored the role of socioeconomic status in the relationship between the daily duration of use and health.

**Results:**

Coefficients were tested using 2-tailed *t* tests, and statistical significance was defined as a 2-sided *P* value less than .05. Quantile regression revealed heterogeneous associations. Compared to nonusers, short-video platform users had better self-rated health at the 70th to 90th quantiles and lower relative health deprivation at the 10th to 30th quantiles. However, the users at the 10th quantile of self-rated health had worse self-rated health (*β*=−2.224, 95% CI −3.835 to −0.613). Longer engagement (≥3 y) correlated with lower relative health deprivation (*β*=1.970, 95% CI 0.308-3.632), while daily use of 1‐4 hours was associated with poorer self-rated health (*β*=−3.385, 95% CI −4.872 to −1.898; *β*=−3.038, 95% CI −5.054 to −1.022) and higher relative health deprivation (*β*=0.035, 95% CI 0.021-0.050; *P*<.001; *β*=0.034, 95% CI 0.014-0.054). Compared to no purposeful use, using with 2 purposes was associated with better self-rated health (*β*=6.082, 95% CI 0.250-11.914) and lower relative health deprivation (*β*=−0.063, 95% CI −0.120 to −0.005). The association was stronger for use with 3 or more purposes. Socioeconomic status moderated the relationship between daily duration of use and health.

**Conclusions:**

This study provides a more specific investigation of how these associations vary across health strata and usage patterns. The findings reveal patterns of benefit and risk across population subgroups, underscoring that how and why individuals engage with platforms matter more than mere access or frequency. These insights necessitate targeted digital well-being policies that protect vulnerable groups, particularly those in poor health or with lower socioeconomic status. Furthermore, policies should actively encourage intentional, functionally grounded use to reduce health inequities and advance equitable digital inclusion.

## Introduction

The rapid proliferation of digital technologies has fundamentally reconfigured human behavior and social dynamics, establishing digital engagement as a pivotal social determinant of health [1]. Emerging evidence suggests that digital engagement patterns exhibit complex relationships with physical and mental well-being [2-6]. Some studies indicate that digital engagement promotes social support and information access [7,8], while others suggest that it correlates with anxiety, depression, and sedentary behavior [9,10]. This paradoxical finding underscores the necessity to consider technological characteristics and user experience variability when assessing the relationship between digital engagement and health.

Among digital platforms, short-video platforms, such as TikTok, Douyin, and Reels, represent a unique research frontier given their algorithmic-driven content delivery, passive consumption modalities, and entertainment-centric design [11]. In China, over 1 billion users engage with short-video content, accounting for 95.2% of internet users as of June 2023 [12]. However, high-frequency passive browsing and performative interactions features of short-video platforms can amplify the potential benefits of digital engagement, as well as exacerbate their risks. These platforms mediate health-related behaviors through 3 intersecting pathways: information, behavior, and psychology. Information pathways primarily shape individuals’ health knowledge and literacy. Studies have shown that internet use for health information was associated with higher health literacy and health service use [13]. But perceived health information overload discourages enhancing health literacy [14]. Behavioral pathways encompass habitual usage behaviors and health-promoting behaviors. Access to health information facilitates the development of health behaviors [15,16], while sedentary behavior and addictive behavior during internet use may adversely affect health [17,18]. Psychological pathways involve platform-based social interactions and psychological symptoms. On the one hand, the internet can expand social networks, providing social support and entertainment [19,20]. On the other hand, problematic internet use may lead to symptoms of anxiety and depression [21]. Crucially, the relationship between these pathways and health is unlikely to be uniform across all individuals but rather varies according to their existing resources and health status.

However, existing studies predominantly focus on aggregate associations between digital engagement and health outcomes. The heterogeneous association between short-video platform use and health regarding different population subgroups remains understudied. One key issue is how its multidimensional characteristics, including the time, frequency, duration, and purpose of short-video platform use, are related to individual health. Moreover, engagement with short-video platforms has been an emerging behavioral determinant nested within broader socioeconomic and environmental structures. The social determinants of health theory holds that health and health inequalities are determined by social, economic, and environmental factors [22]. The digital health paradox further sharpens this lens, highlighting that people excluded by digital technology face the risk of being abandoned by society in most cases. So, people who have very limited access to the internet are precisely those who need the most health services and bear the heaviest health burden, such as low-income populations and long-term ill individuals [23]. Therefore, it is necessary to consider whether the association between short-video platform use and health exhibits heterogeneity across different health distributions and socioeconomic status (SES) groups.

To comprehensively understand the relationship between short-video platform use and individual health, the measurement of health also requires a multidimensional perspective. Self-rated health has been validated to comprehensively reflect overall health status and effectively predict mortality [24], yet it fails to capture the distribution and accumulation of health conditions. To address this limitation, we incorporate relative health deprivation, operationalized through the Kakwani index [25]. Relative deprivation theory holds that an individual’s health level is negatively correlated with health disadvantages within a specific group, which means the lower the health level, the greater the health disadvantage and the higher the degree of relative deprivation of health faced. By calculating the health deprivation index, it is possible to reflect the long-term and structural accumulation of health disadvantages.

This study aims to investigate the heterogeneous associations between short-video platform use and individual health across different health status and usage behaviors. Specifically, we aim to (1) examine the association between whether using short-video platform and health varies across the health distribution, (2) analyze the associations between multidimensional usage behaviors (years, frequency, daily duration, and purpose diversity) and health, and (3) explore the moderating role of SES in the relationship between daily duration of use and health. By identifying vulnerable subgroups involved in digital participation, this study provides critical, actionable insights for designing targeted digital health interventions and equitable platform governance policies.

## Methods

### Inclusion and Exclusion

Individuals who met the following criteria were included in this survey: (1) 18 years of age or older and (2) were able to communicate effectively with investigators. The exclusion criteria included (1) no cognitive disorders and (2) no mental disturbance. The data were collected using a structured questionnaire. Questionnaires with completion times below 5 minutes or above 50 minutes were excluded, as pilot testing indicated these thresholds captured inattentive or interrupted responses. Questionnaires with a response missing rate exceeding 50% for the purpose of quality control were also excluded.

### Participant Characteristics

Of the 7725 participants included in the final analysis, 3628 (46.96%) were male, and the average age was 65.49 (SD 9.39) years. A total of 4403 (57.0%) participants were users of short-video platforms. Further detailed characteristics of the participants, including demographic, socioeconomic, health-related, and social support factors, are presented in Table 1.

**Table 1.** Characteristics of the participants before propensity matching.

Characteristics	All	Short-video platforms		Self-rated health		Relative health deprivation	
Users	Nonusers	Mean (SD)	*P* value	Mean (SD)	*P* value
N	7725 (100)	4403 (57.00)	3322 (43.00)	—^a^	—	—	—
Age (y), mean (SD)	65.49 (9.39)	61.92 (8.68)	70.21 (8.12)	65.59 (17.17)	<.001	0.14 (0.17)	<.001
Sex, n (%)					.007		.09
Male	3628 (46.96)	2270 (51.56)	1358 (40.88)	66.15 (17.40)		0.14 (0.17)	
Female	4097 (53.04)	2133 (48.44)	1964 (59.12)	65.09 (16.96)		0.15 (0.17)	
Marriage, n (%)					<.001		<.001
Married or cohabiting	6492 (84.04)	3966 (90.07)	2526 (76.04)	66.17 (16.98)		0.14 (0.16)	
Other marital status^b^	1233 (15.96)	437 (9.93)	796 (23.96)	62.52 (17.85)		0.17 (0.18)	
Urban-rural residence, n (%)					<.001		<.001
Urban	2342 (30.32)	1332 (30.25)	1010 (30.40)	67.93 (16.02)		0.12 (0.15)	
Rural	5383 (69.68)	3071 (69.75)	2312 (69.60)	65.59 (17.56)		0.15 (0.17)	
Socioeconomic status, n (%)					<.001		<.001
Low	2880 (37.28)	1622 (36.84)	1258 (37.87)	61.07 (18.19)		0.19 (0.19)	
Middle	4310 (55.79)	2416 (54.87)	1894 (57.01)	68.00 (15.86)		0.12 (0.14)	
High	535 (6.93)	365 (8.29)	170 (5.12)	70.39 (16.49)		0.11 (0.15)	
Education, n (%)					<.001		<.001
Illiterate or semiliterate	2960 (38.32)	1104 (25.07)	1856 (55.87)	63.45 (16.86)		0.16 (0.17)	
Primary school	2277 (29.48)	1336 (30.34)	941 (28.33)	65.42 (17.56)		0.15 (0.17)	
Middle school	1619 (20.96)	1281 (29.09)	338 (10.17)	67.46 (16.96)		0.13 (0.16)	
High school or vocational school	686 (8.88)	535 (12.15)	151 (4.55)	69.40 (16.72)		0.11 (0.16)	
College degree / Bachelor's degree or above	183 (2.37)	147 (3.34)	36 (1.08)	71.30 (15.26)		0.10 (0.14)	
Employment status, n (%)					<.001		<.001
Unemployed	4539 (58.76)	2337 (53.08)	2202 (66.29)	64.22 (17.51)		0.16 (0.18)	
Employed	3186 (41.24)	2066 (46.92)	1120 (33.71)	67.53 (16.49)		0.13 (0.15)	
Convenience of obtaining medical services, n (%)					<.001		<.001
Low	22 (0.29)	15 (0.34)	7 (0.21)	55.18 (27.16)		0.28 (0.32)	
Medium low	284 (3.68)	104 (2.36)	180 (5.42)	55.10 (19.23)		0.25 (0.21)	
Middle	900 (11.67)	378 (8.59)	522 (15.71)	59.49 (18.08)		0.20 (0.19)	
Medium high	4394 (57.00)	2448 (55.60)	1946 (58.58)	66.55 (16.46)		0.13 (0.16)	
High	2109 (27.36)	1458 (33.11)	667 (20.08)	67.74 (16.72)		0.13 (0.16)	
Number of persons living together, n (%)					.008		<.001
0	740 (9.58)	319 (7.25)	421 (12.67)	63.92 (16.88)		0.16 (0.17)	
1-3	3961 (51.28)	2331 (52.94)	1630 (49.07)	65.22 (17.48)		0.15 (0.17)	
4-6	2657 (34.39)	1543 (35.04)	1114 (33.53)	66.36 (16.96)		0.14 (0.16)	
>6	367 (4.75)	210 (4.77)	157 (4.73)	67.34 (15.42)		0.12 (0.14)	
Number of persons contacting with regularly, n (%)					<.001		<.001
0	307 (3.97)	151 (3.43)	156 (4.70)	58.57 (19.56)		0.22 (0.21)	
1	1348 (17.45)	583 (13.24)	765 (23.03)	64.94 (17.34)		0.15 (0.18)	
2-3	2855 (3.96)	1600 (36.34)	1255 (37.78)	65.76 (1660)		0.14 (0.16)	
4-5	1567 (20.28)	915 (20.78)	652 (19.63)	66.44 (15.91)		0.13 (0.15)	
≥6	1648 (21.33)	1154 (26.21)	494 (14.87)	66.31 (18.36)		0.14 (0.18)	
Multimorbidity-weighted index, mean (SD)	2.51 (2.02)	2.40 (1.96)	2.66 (2.09)	65.59 (17.17)	<.001	0.14 (0.17)	<.001
Depression, mean (SD)	5.87 (4.78)	5.28 (4.50)	6.65 (5.02)	65.59 (17.17)	<.001	0.14 (0.17)	<.001
Anxiety, mean (SD)	2.07 (3.45)	1.82 (3.22)	2.39 (3.71)	65.59 (17.17)	<.001	0.14 (0.17)	<.001
Loneliness, mean (SD)	3.70 (1.29)	3.54 (1.19)	3.90 (1.39)	65.59 (17.17)	<.001	0.14 (0.17)	<.001

a Not applicable.

b Other marital status includes being widowed, separated, divorced, or never married.

### Sampling Procedures

Multistage stratified sampling was applied to select participants in the survey from July to September 2024. First, initial stratification was conducted based on geographical region. Fujian province, Hubei province, and Chongqing municipality were chosen as the survey areas, which are respectively located in the eastern, central, and western regions of China. Second, within each province, 6‐12 urban subdistricts and rural townships were randomly selected as secondary sampling units, ultimately covering 24 subdistricts or townships. Finally, through the community registries, 300‐400 participants were randomly selected from each subdistrict or township. Sampling weights were not applied in the analyses, and all estimates are presented as unweighted statistics. The sampling design aimed to ensure structural representativeness across regional and urban-rural strata within the selected provinces. The data were collected through face-to-face interviews with the respondents by uniformly trained investigators, and the questionnaires were filled out simultaneously. This study was conducted and reported following the Journal Article Reporting Standards for quantitative research [26].

### Sample Size, Power, and Precision

A total of 7725 valid samples were included from the survey, yielding an effective recovery rate of 85.48%. All the analyses were conducted on this sample of valid questionnaires, and there were no missing data for the variables required by this study. Sample size estimation followed the standard formula for proportion-based calculations: [$n=\left(u_{\alpha }^{2}pq\right)∕d^{2}$], where $u_{\alpha }$ represents the critical value for a 95% CI ($u_{\alpha }$=1.96), *p* is the estimated proportion of short-video platform users based on the 52nd Statistical Report on Internet Development in China—Research on Internet Development [12], *q* is the complementary proportion (q=1-p), and d denotes the allowable error (1.2%). Using these parameters, the minimum required sample size was calculated as 5283.

### Measures and Covariates

#### Self-Rated Health

Self-rated health was applied to reflect an individual’s health status. Self-rated health was assessed using a visual analog scale. Participants were asked the following question: “Overall, how would you rate your current health status?“ They responded by selecting a point on a horizontal scale ranging from 0 to 100, with a higher score indicating a better level of health.

#### Relative Health Deprivation

We measured relative health deprivation by calculating the Kakwani relative deprivation index using individuals’ self-rated health, referring to the research of Kakwani [25]. The first step is to assume *G* as a reference group containing *k* sample sizes and rank the self-rated health levels of individuals in this group in ascending order. Then we can obtain *G* = (*g*_1_,*g*_2_,*g*_3_, … ,*g*_*k*-1_,*g*_*k*_), which is the distribution vector of self-rated health. Therefore, compared with the self-rated health of individual *i* with that of *j*, the relative health deprivation of individual *i* is as follows:


(1) $$RD(g_{j}-g_{i})=\left\{ \begin{array}{ll} g_{j}-g_{i} & \text{if }g_{j}\geq g_{i}\\ 0 & \text{if }g_{j}§lt;g_{i} \end{array}\right.$$

On this basis, the average relative health deprivation for individual *i* can be calculated as follows:


(2) $$RD(g_{i})=\frac{1}{k\lambda_{G}}\left(k_{g}^{+}g_{i}^{+}-k_{g}^{+}g_{i}\right)$$

where *λ*_*G*_ is the average self-rated health of all samples within the reference group *G*. ***${\text{k}}_{{\text{g}}_{\text{i}}}^{\text{+}}$*** is the number of individuals whose self-rated health exceeds that of individual *i*. $\lambda_{g_{i}}^{+}$ is the average self-rated health of individuals who have higher self-rated health than *i*.

The reference group for calculating relative health deprivation was defined as all participants within the same geographically stratified sampling group. This allows the deprivation measure to reflect within-region health comparisons.

#### Short-Video Platform Use

We took “whether to use short video software” as one of the independent variables, with the options containing yes and no. Besides, due to the multidimensional characteristics of digital engagement, years, frequency, daily duration, and purpose diversity of short-video platform use were selected to measure the usage patterns. The assessment methods for the four dimensions are as follows: (1) years of use included “≤1 year,” “1-3 years,” and “≥3 years,” with the assigned values being 1, 2, and 3; (2) frequency of use included “several times a year,” “several times a month,” “1-4 times a week,” and “almost every day,” with values ranging from 1 to 4; (3) daily duration of use included: “≤30 minutes,” “31-60 minutes,” “61-120 minutes,” “120-240 minutes,” and “≥240 minutes,” with values ranging from 1 to 5; and (4) the purpose diversity of use mainly measures the number of intended uses, regarding information acquisition, entertainment, social interaction, creative sharing, and economic acquisition. The options of number of purposes included “0,” “1,” “2,” and “≥3,” with values ranging from 1 to 4.

#### SES

To further analyze the role of socioeconomic factors in the relationship between short-video platform use and health, SES was incorporated into the moderating effect analysis, and it was set at 3 levels: low, middle, and high. The SES level was reported by the participants themselves.

#### Covariates

We used a series of demographic and socioeconomic factors, social support factors, and health-related factors as covariates. Demographic and socioeconomic factors included age, gender, urban-rural residence, SES, education, and employment status. Social support factors measured marriage, number of persons living together, and number of persons contacted regularly, following the framework proposed by van den Brink et al [27]. Among health-related factors, the Chinese multimorbidity–weighted index was used to control multimorbidity burden, which was calculated according to Hu et al [28]. Moreover, the convenience of obtaining medical services and mental health, including depression (as assessed with 10-item Center for Epidemiologic Studies Depression Scale) [29], anxiety (as assessed with 7-item Generalized Anxiety Disorder) [30], and loneliness (as assessed with University of California at Los Angeles Loneliness Scale) [31], was also controlled. The options included by these variables are shown in Table 1.

### Statistical Analysis

First, 2-tailed *t* test and variance analysis were used to analyze the differences in self-rated health and relative health deprivation among different groups. In the second step, propensity score matching analysis with no replacement was performed to control for selection bias based on the probability of short-video platform use (users and nonusers). Afterward, we fitted quantile regression models on the 10th, 20th, 30th, 40th, 50th, 60th, 70th, 80th, and 90th quantiles of the self-rated health and relative health deprivation distribution to investigate the differences in health between short-video platform users and nonusers. Quantile indicates the location of individuals within the outcome distribution [32]. For example, the individual in the 80th quantile of the conditional distribution has a self-rated health larger than 79% of those with similar short-video platform use behaviors. Quantile regression is a flexible and powerful tool that can assess how health relates to short-video platform use at any quantile distribution. Then, a linear regression model was applied to explore the association of different dimensions of short-video platform use with health, using the full sample. Independent variables consisted of the years, frequency, daily duration, and purpose diversity of use. Finally, we used moderating effect analysis to explore the moderating role of SES between the daily duration of use and health. A series of sensitivity analyses was conducted to test the robustness of the results. Quantile regression was conducted again based on propensity score matching with replacement. In the linear regression model, years, frequency, daily duration, and purpose diversity of use were regarded as continuous variables to be analyzed. Further, we limited the analysis of these dimensions to users of short-video platforms, so as to make the coverage more targeted. The urban-rural residence was used to replace the self-assessed SES to evaluate the moderating role of socioeconomic factors. In all models, coefficients were tested using 2-tailed *t* tests.

Stata 18.0 was used for all analyses. Statistical significance was defined as a 2-sided *P* value less than .05.

### Ethical Considerations

#### Ethics Review and Approval

This study involved human participants and was conducted in accordance with the Declaration of Helsinki. The study protocol was reviewed and approved by the Medical Ethics Committee of Tongji Medical College, Huazhong University of Science and Technology (approval No. [2023] Lun Shen Zi (S193)).

#### Informed Consent

Prior to participation, all individuals were provided with detailed information about the study’s purpose, procedures, potential risks, and benefits. Written informed consent was obtained from each participant.

#### Privacy and Confidentiality

To protect participant privacy, all collected data were anonymized at the point of collection. Personal identifiers were removed, and each participant was assigned a unique study code. The data were stored on password-protected secure servers accessible only to the authorized research team.

#### Participant Compensation

Participants did not receive financial compensation for participation. However, small tokens of appreciation (toothpaste of RMB ¥10 [approximately US $1.42]) were provided in accordance with local ethical norms to acknowledge participants’ time.

#### Image and Identifiable Data Protection

This manuscript and its supplementary materials do not contain any images, videos, or personal details that could lead to the identification of an individual participant.

## Results

### Characteristics of Participants

**Figure 1.**
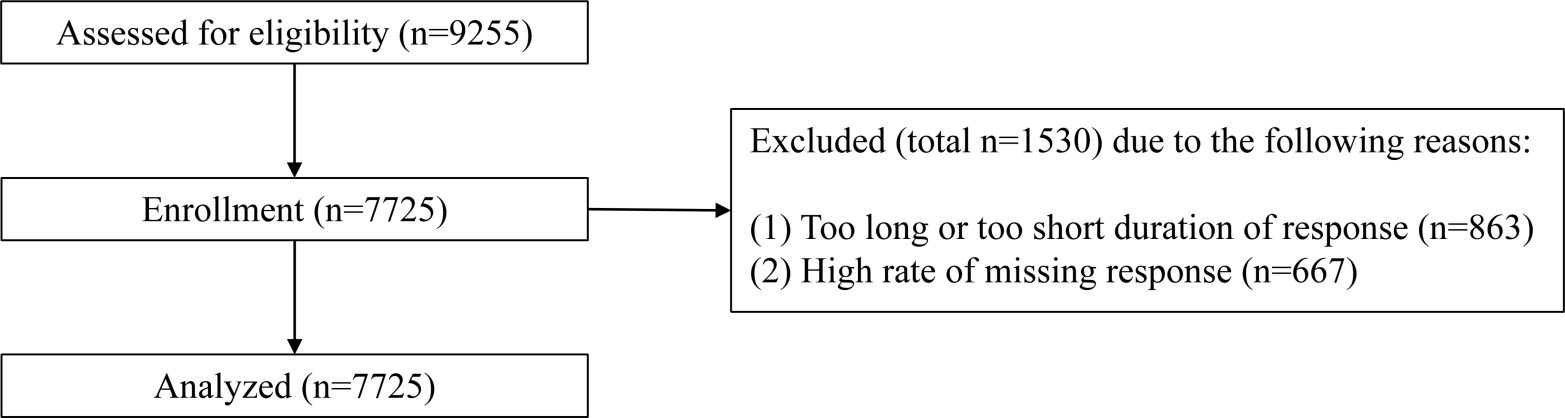
Flow diagram of participant recruitment and data exclusion.

The final sample consisted of 7725 participants in this study (Figure 1). Characteristics of eligible participants are presented in Table 1. Of the 7725 participants, 4403 (57%) used short-video platforms, 3628 (46.96%) were male, 6492 (84.04%) were married or cohabiting, and 2342 (30.32%) lived in urban areas. Participants who were spousal (n=3966, 90.07% vs n=2526, 76.04%) and had received education (n=6621, 74.93% vs n=5869, 44.13%) were more likely to use short-video platforms. Compared with nonusers of short-video platforms, users had lower scores of multimorbidity-weighted index (2.40, SD 1.96 vs 2.66, SD 2.09), depression (5.28, SD 4.50 vs 6.65, SD 5.02), anxiety (1.82, SD 3.22 vs 2.39, SD 3.71), and loneliness (3.54, SD 1.19 vs 3.90, SD 1.39). The differences in scores of self-rated health and relative health deprivation among different groups are shown in Table 1.

### Quantile Regression Results

Figures S1-S2 in Multimedia Appendix 1 show the distribution of self-rated health and relative health deprivation in quantiles. The 2 purple curves fluctuate to a large extent near the gray uniform line, indicating that the scores of self-rated health and relative health deprivation are not normally distributed. So, it is necessary to apply quantile regression. In total, 3322 pairs of users of short-video platforms versus nonusers of short-video platforms were formed based on the propensity matching scores. The characteristics of the participants in the propensity-matched dataset are provided in Table S1 in Multimedia Appendix 1.

The quantile regression results in the propensity-matched dataset are reported in Table 2. Compared with the nonusers of short-video platforms, the self-rated health of users was higher at the 70th to 90th quantile (*P*<.05), and the score of the relative health deprivation of users was lower at the 10th to 30th quantile (*P*<.05). For self-rated health, the correlation was the strongest at the 90th quantile (*β*=1.931, 95% CI 1.299-2.563; *P*<.001). Compared to the users at the 20th and 30th quantiles, the score of relative health deprivation decreased to a lesser extent among those at the 10th percentile (*β*=−0.003, 95% CI −0.005 to −0.001; *P*<.001). Moreover, the self-rated health of users is lower than nonusers below the 10th quantile (*β*=−2.224, 95% CI −3.835 to −0.613; *P*=.007), while no significant association was observed at the 90th quantile of relative health deprivation. There was no significant association at the 20th to 60th quantile of self-rated health and the 40th to 90th quantile of relative health deprivation.

**Table 2.** Results for quantile regression analysis of the association between the use of short-video platforms and self-rated health, and relative health deprivation in propensity-matched dataset.

Quantile (reference: nonusers)	Self-rated health^a^		Relative health deprivation^b^	
	Coefficient (95% CI)	*P* value	Coefficient (95% CI)	*P* value
0.1	–2.224 (–3.835 to –0.613)	.007	–0.003 (–0.005 to –0.001)	<.001
0.2	–0.013 (–1.375 to 1.348)	.99	–0.005 (–0.007 to –0.002)	.001
0.3	0.813 (–0.355 to 1.982)	.17	–0.005 (–0.007 to –0.002)	<.001
0.4	0.544 (–0.521 to 1.608)	.32	–0.002 (–0.007 to 0.003)	.38
0.5	0.772 (–0.600 to 2.145)	.27	–0.001 (–0.006 to 0.003)	.58
0.6	0.627 (–0.304 to 1.558)	.19	–0.005 (–0.012 to 0.002)	.15
0.7	0.990 (0.067 to 1.913)	.04	–0.008 (–0.020 to 0.003)	.15
0.8	1.423 (0.600 to 2.246)	.001	–0.002 (–0.019 to 0.014)	.78
0.9	1.931 (1.299 to 2.563)	<.001	0.021 (–0.001 to 0.043)	.07

a Adjusted *R*2: 0.179; *F*-value: 97.67; *F* significance: <.001.

b Adjusted *R*2: 0.17; *F*-value: 91.78; *F* significance: <.001

### Linear Regression Results

Correlation analysis indicated that there was a correlation among years, frequency, daily duration, and the number of use purposes of short-video platforms usage. The results of the multicollinearity test showed that there was no collinearity among these variables (variance inflation factor<5). The specific results are shown in Tables S2-S3 in Multimedia Appendix 1.

As shown in Table 3, compared with participants who have used short-video platforms for ≤1 year, those who have used them for ≥3 years showed significantly better self-rated health (*β*=1.970, 95% CI 0.308-3.632; *P*<.05). Yet, after adjusting for the control variables, there was no significant correlation between the years of use and relative health deprivation. There was no significant correlation between frequency of use and self-rated health and relative health deprivation. Participants who spent 61‐120 minutes and 120‐240 minutes per day had significantly worse self-rated health (*β*=−3.385, 95% CI −4.872 to −1.898; *P*<.001; *β*=−3.038, 95% CI −5.054 to −1.022; *P*<.01) and higher score of relative health deprivation (*β*=0.035, 95% CI 0.021-0.050; *P*<.001; *β*=0.034, 95% CI 0.014-0.054; *P*<.01). However, there was no significant relationship between the participants’ daily duration of use exceeding 240 minutes and their self-rated health or relative health deprivation. Compared to no purposeful use, using short-video platforms for 2 purposes was associated with better self-rated health (*β*=6.082, 95% CI 0.250-11.914; *P*<.05) and lower relative health deprivation (*β*=−0.063, 95% CI −0.120 to −0.005; *P*<.05). The association was stronger for use with ≥3 purposes (self-rated health: *β*=6.213, 95% CI 0.276-12.151; *P*<.05; relative health deprivation: *β*=−.068, 95% CI −0.127 to −0.009; *P*<.05).

**Table 3.** Association between the usage behaviors of short-video platforms and self-rated health and relative health deprivation.

Variables	Self-rated health		Relative health deprivation	
Model 1, coefficient (95% CI)	Model 2, coefficient (95% CI)	Model 3, coefficient (95% CI)	Model 4, coefficient (95% CI)
Years of use (reference: ≤1)				
1-3	1.775 (–0.012 to 3.562)	1.156 (–0.493 to 2.805)	–0.011 (–0.029 to 0.006)	–0.006 (–0.023 to 0.010)
≥3	4.116 (2.332 to 5.900)^a^	1.970 (0.308 to 3.632)^b^	–0.032 (–0.050 to –0.015)^a^	–0.014 (–0.031 to 0.002)
Frequency of use (reference: Several times a year)				
Several times a month	–4.718 (–11.378 to 1.941)	–5.033 (–11.13 to 1.074)	0.050 (–0.016 to 0.115)	0.053 (–0.007 to 0.114)
1-4 times a week	–4.399 (–10.485 to 1.687)	–4.673 (–10.255 to 0.907)	0.039 (–0.020 to 0.099)	0.043 (–0.012 to 0.098)
Almost every day	–4.983 (–10.997 to 1.032)	–5.250 (–10.763 to 0.262)	0.049 (–0.010 to 0.108)	0.052 (–0.002 to 0.107)
Daily duration of use (min; reference: ≤30)				
31-60	1.021 (–0.396 to 2.437)	0.046 (–1.259 to 1.350)	–0.008 (–0.022 to 0.006)	0.002 (–0.011 to 0.015)
61-120	–2.451 (–4.067 to –0.834)^c^	–3.385 (–4.872 to –1.898)^a^	0.026 (0.010 to 0.042)^c^	0.035 (0.021 to 0.050)^a^
120-240	–2.856 (–5.055, –0.657)^b^	–3.038 (–5.054 to –1.022)^c^	0.033 (0.011 to 0.054)^c^	0.034 (0.014 to 0.054)^c^
≥240	0.202 (–3.220 to 2.815)	–0.352 (–3.119 to 2.415)	0.008 (–0.022 to 0.038)	0.009 (–0.018 to 0.037)
Numbers of purposes of use (reference: 0)				
1	5.694 (–0.564 to 11.953)	4.970 (–0.773 to 10.712)	–0.055 (–0.117 to 0.006)	–0.051 (–0.108 to 0.006)
2	7.263 (0.910 to 13.616)^b^	6.082 (0.250 to 11.914)^b^	–0.071 (–0.133 to –0.008)^b^	–0.063 (–0.120 to –0.005)^b^
≥3	8.349 (1.890 to 14.807)^b^	6.213 (0.276 to 12.151)^b^	–0.084 (–0.147 to –0.020)^b^	–0.068 (–0.127 to –0.009)^b^
Control variables	No	Yes	No	Yes
Adjusted *R*^2^	0.021	0.18	0.016	0.166
*F*-value	14.82	66.1	11.21	59.95
*F* significance	<.001	<.001	<.001	<.001

a *P*<.001.

b *P*<.05.

c *P*<.01.

### Moderating Effect Analysis Results

**Figure 2.**
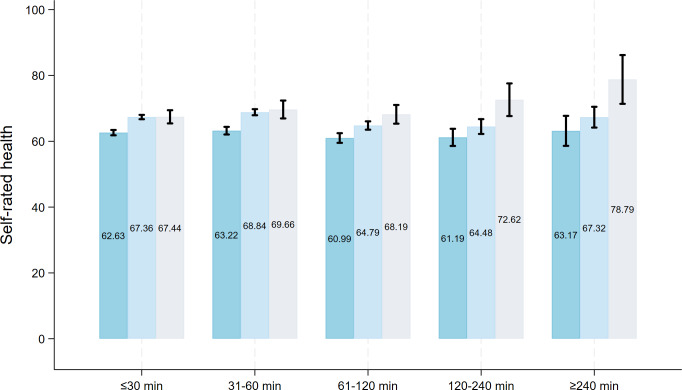
Results for moderating effect analysis between daily duration of short-video platforms usage and self-rated health. “≤30 minutes,” “31-60 minutes,” “61-120 minutes,” “120-240 minutes,” and “≥240 minutes” indicate daily duration of use. Error bars represent 95% CI.

As shown in Figures2  3, SES was a significant moderator of the association between daily duration of use and health (all *P* for interaction <.05). The corresponding coefficients and *P* for interaction can be found in Table S4 in Multimedia Appendix 1. Specifically, compared to the high SES group, participants with middle SES showed significantly lower self-rated health (*β*=−2.257, 95% CI −3.540 to −0.975; *P*=.001) and higher relative health deprivation (*β*=0.018, 95% CI 0.005-0.031; *P*=.005). Similarly, the low SES group showed significantly poorer self-rated health (*β*=−2.022, 95% CI −3.349 to −0.695; *P*=.003) and a higher score of health deprivation (*β*=0.014, 95% CI 0.001-0.027; *P*=.04).

**Figure 3.**
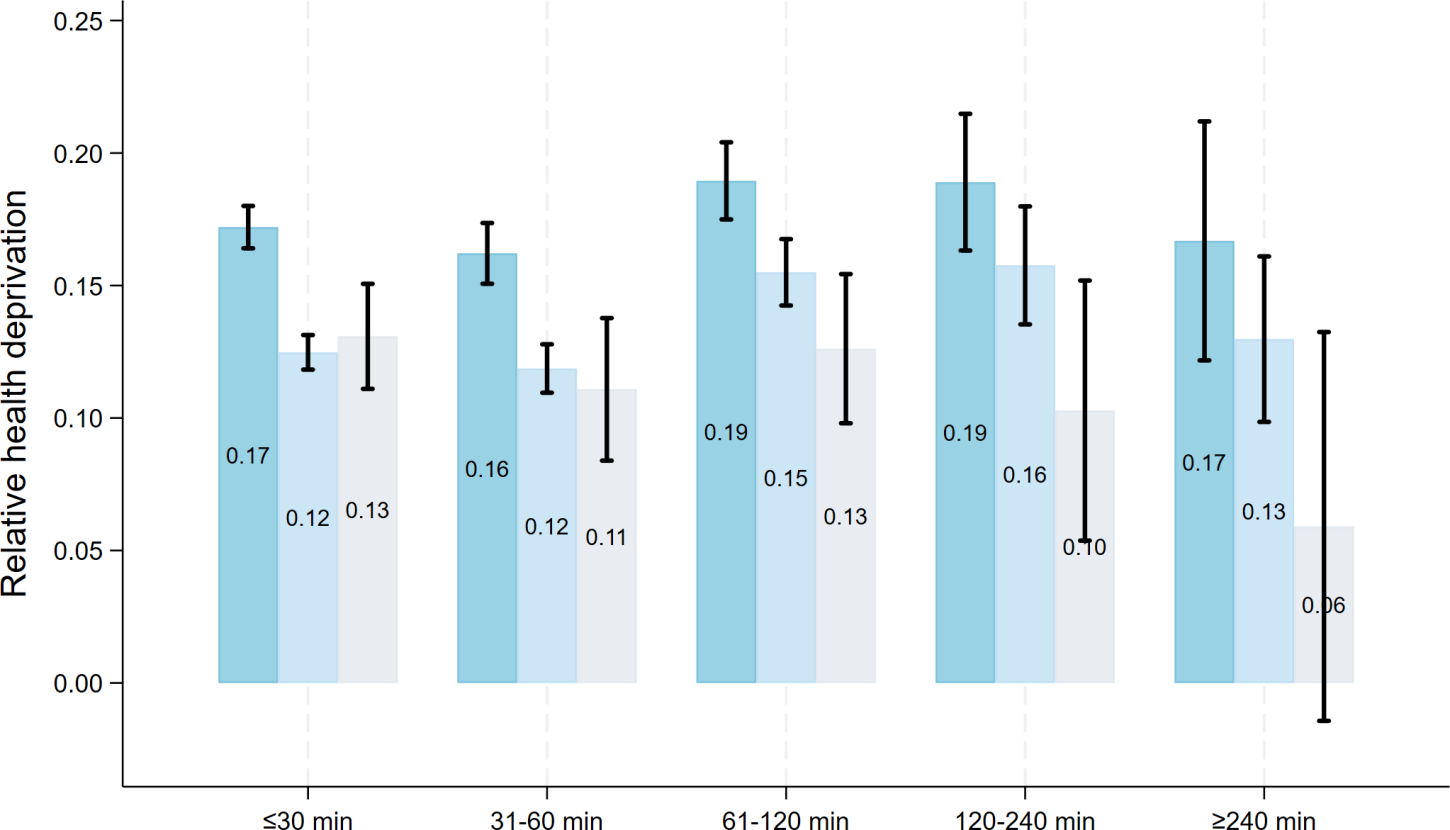
Results for moderating effect analysis between daily duration of short-video platforms usage and relative health deprivation. “≤30 minutes,” “31-60 minutes,” “61-120 minutes,” “120-240 minutes,” and “≥240 minutes” indicate daily duration of use. Error bars represent 95% CI.

### Sensitivity Analysis Results

After changing the propensity score matching method, the conclusions of the quantile regression remained unchanged. Regarding years, frequency, daily duration, and purpose diversity of usage as continuous variables in linear regression, the relevant conclusions are broadly consistent. The analysis results for the users of short-video platforms also found similar results. Moreover, compared to the urban residence group, the rural residence group had significantly lower self-rated health (*β*=−0.890, 95% CI −1.667 to −.113; *P*=0.03) and higher relative health deprivation (*β*=0.008, 95% CI 0.000-0.016; *P*=.038). This is also consistent with the previous analysis of the moderating role. All sensitivity analysis results are provided in Tables S5-S8 in Multimedia Appendix 1.

## Discussion

### Principal Results

This study explored the heterogeneous association between multidimensional short-video platform use and individual health among Chinese populations. The results reveal a series of nuanced findings. Only among the health-advantaged group with relatively high self-rated health (70th-90th quantile) and low relative deprivation (10th-30th quantile), using short-video platforms was associated with better self-rated health and lower relative health deprivation compared to nonuse. Moreover, short-video platform use was linked to worse self-rated health in the least healthy group (10th percentile) yet showed no significant association with health deprivation in the most relatively deprived group (90th percentile). Another finding showed that greater purpose diversity (≥2 purposes) and longer engagement (≥3 y) with short-video platforms were positively associated with better self-rated health. However, frequency of use showed no significant link to health. A daily use duration of 1 to 4 hours was associated with poorer self-rated health and higher relative deprivation, yet no such negative association was found for duration exceeding 4 hours. SES significantly moderated the association between daily use duration and relative health deprivation, with the strength of this association being more pronounced among middle- and low-SES groups than in the high-SES group.

### Interpretation

Our findings demonstrate theoretical congruence with the diffusion of innovations framework. This theory posits that technology adoption follows a social gradient, often privileging early adopters who possess greater resources [33]. The health-advantaged group has preexisting health and social resources that are better equipped to navigate digital environments selectively. They can make use of these advantages for information, social connection, and stress relief, thereby further enhancing their well-being [34]. Their lower baseline deprivation may also buffer them against negative social comparison, a known risk of short-video platforms use. Conversely, the significant negative association observed in the least healthy group (10th percentile of self-rated health) suggests potential pathways of harm. Individuals in poor health may be more vulnerable to algorithmic traps and engage in passive consumption of content. Their use may exacerbate sedentary behavior or increase exposure to distressing or misleading health information [35-37]. Another noteworthy finding is that there is no significant correlation between short-video platform use and relative health deprivation among the group with the highest level of health deprivation (10th percentile). It indicates that for individuals who perceive themselves as severely disadvantaged compared to others, engagement with short-video platforms does little to relieve this sense of inequity [38]. Their deprivation likely depends on structural factors, such as the severity of the disease and socioeconomic disadvantage. These cannot be easily relieved by the fragmented social support and information on short-video platforms. For individuals at the 20th to 60th percentiles of self-rated health and at the 40th to 90th percentiles of relative health deprivation, no significant association was observed. This group typically fits the description of the early majority as defined in diffusion of innovations theory [33]. Their habits of use are more likely to be influenced by the inherent characteristics of short videos, which are typically characterized by frequent but brief, passive, and entertainment-oriented consumption [39]. Together, this pattern of use tends to make short videos a relatively insignificant supplementary tool. Its association with cognitive, emotional, and behavioral remains diffuse and thus below the threshold of detectable statistical association in a cross-sectional design. Notably, our finding of a modest reduction in relative health deprivation (*β*=−0.003) at the 10th percentile requires careful interpretation. While this change is small at the individual level, its consistent detection at the lower tail of the deprivation distribution holds broader significance. It suggests that even among those who already feel relatively less deprived, short-video platform use may contribute a marginal, yet statistically robust, psychological sense of gain. From a population health perspective, a small shift that is reliably present across a large subgroup can translate into a meaningful aggregate benefit [40].

The differentiated associations observed between behaviors of short-video platform use and health suggest that how and why individuals engage with these platforms may matter more than how often they do so. The positive association of purpose diversity (≥2 purposes) with health underscores the importance of an active, agentic attitude for short-video platforms use. Short-video platforms are engineered for high engagement through infinite scroll and personalized feeds, which typically promote passive viewing [41]. Users who could actively use the platform for information seeking, social interaction, and content creation likely mitigate risks of mindless scrolling [42-44]. The association linked to longer engagement (≥3 y) may reflect digital literacy increases with the accumulation of use time. New users need to spend time exploring and mastering the functions of the short-video platforms, while long-term users may develop more regular and proactive habits. They might be able to use the platform’s functions more flexibly and benefit from it [45,46]. No significant correlation between frequency of use and health is observed, which is inconsistent with the previous studies regarding internet usage [10,47,48]. It might be due to the inherent characteristics of the short-video platforms. Unlike traditional media consumption, where each session constitutes a discrete, sustained period of attention, short-video use is characterized by brief and repetitive engagements [49]. Therefore, the frequency of use may not accurately reflect its association with health, as it fails to distinguish between these short usage periods and more prolonged, immersive usage periods. For the daily duration of use, using short-video platforms for 1 to 4 hours per day is associated with poorer health. This might indicate the psychological fatigue caused by excessive use and the adverse physical activities, such as sedentary behavior [5,50,51]. The daily duration of use exceeding 4 hours was not significantly associated with self-rated health and relative health deprivation. This might be related to the user’s occupation, pattern of use, and so on. This requires further analysis in subsequent studies.

The moderating role of SES observed in this study also aligns with the innovation diffusion theory and the digital divide theory [33]. From the perspective of innovation diffusion, high-SES individuals often act as early adopters. Their greater material and cognitive resources facilitate not only access but also more adaptive and beneficial engagement with new technologies, such as short-video platforms. This translates into higher digital health literacy, better self-regulatory capacity, and the ability to use these platforms more selectively [52-54]. Conversely, the theory of the second-level digital divide explains the heightened vulnerability of middle- and low-SES groups [55]. Disparities in digital skills and resources often confine their engagement to more passive, entertainment-focused consumption patterns [56]. This pattern of use, characterized by prolonged but less intentional exposure, makes them more susceptible to the platforms’ risks, such as unfavorable social comparison, algorithmic traps, and sedentary behavior [57]. Thus, preexisting socioeconomic inequalities are reproduced and potentially amplified in the digital realm, exacerbating feelings of relative health deprivation among the already disadvantaged.

### Limitations

Several limitations of this study should be acknowledged. First, despite the application of propensity score matching to mitigate selection bias, the inherent constraints of cross-sectional observational design preclude definitive causal inferences regarding short-video platform use and health outcomes [24]. Second, while multistage stratified sampling was implemented to ensure regional diversity, potential sampling biases may exist due to nonprobability sampling methods and self-reported participation rates, limiting the generalizability of the findings to the broader Chinese adult population. Third, self-rated health as a subjective measure is susceptible to social desirability bias and cultural interpretation variance, potentially underestimating or overestimating actual health disparities. Fourth, although multiple covariates were statistically controlled, residual confounding effects from unmeasured variables, such as cognitive function and digital literacy, cannot be entirely ruled out, particularly given their demonstrated associations with both health behaviors and technology engagement patterns. Fifth, SES relying on self-report may have measurement error. Future studies would benefit from incorporating objective indicators, such as household income brackets, educational attainment levels, and occupational prestige scores to enhance construct validity. Sixth, the categorization of screen time into discrete intervals limits our ability to model dose-response relationships or detect nonlinear threshold effects within usage categories. This limitation could be addressed through device-based time-use monitoring in subsequent research. Seven, the lack of content type differentiation obscures the understanding of how specific platform features link to health, an issue that could be resolved through qualitative content analysis. Finally, the study focused on short-video platforms and did not capture the potential association between other digital media forms and health. Future longitudinal studies should incorporate specific contents of usage to better identify for whom and under what conditions digital engagement supports health.

### Conclusions

This study advances the field by moving beyond the examination of overall associations in the novel context of short-video platforms. It investigates for whom and under what conditions the use of these platforms is linked to health. By exploring multidimensional usage behaviors, it identifies distinct patterns of benefit and risk across population subgroups, providing a nuanced understanding of the relationship between digital engagement and health. Self-rated health and relative health deprivation help to clarify an individual’s subjective perception of health, as well as the sense of health inequality derived from social comparisons. This study carries significant associations between short-video platform use and individual health, which are heterogeneous across population subgroups. Policy must therefore shift from ensuring universal access to actively governing for equitable digital well-being. This requires targeted protections for vulnerable groups, especially those in poor health or with lower SES. They are most susceptible to harm from passive or prolonged use. Meanwhile, the observed disparities in relative health deprivation suggest public policy should aim not only to improve material health access but also to intentionally shape digital environments to address both material and perceived health disparities.

## Supplementary material

10.2196/86526 Multimedia Appendix 1 Additional results.

## Abbreviations

SES: socioeconomic status
