# Supplementary material for: Associations Between Short-Video Platform Use and Health Across Health Distribution and Usage Behaviors in China: Cross-Sectional Questionnaire Study
Source: J Med Internet Res. 2026 Mar 12;28:e86526. doi: 10.2196/86526 (PMC12982707; doi:10.2196/86526)
Supplement: Multimedia Appendix 1 [file jmir-v28-e86526-s001.docx]

**Additional Ma****terials**

contents

**[Figures](#_Toc9261)** [2](#_Toc9261)

**[Figure S1. The data distribution of self-rated health.](#_Toc25005)** [2](#_Toc25005)

**[Figure S2. The data distribution of relative health deprivation.](#_Toc14279)** [2](#_Toc14279)

**[Tables](#_Toc21082)** [3](#_Toc21082)

**[Table S1. Characteristics of the participants in propensity-matched dataset.](#_Toc4591)** [3](#_Toc4591)

**[Table S2. Correlation analysis of the usage behaviors of short-video platforms.](#_Toc13175)** [5](#_Toc13175)

**[Table S3. Test for multicollinearity of the years, frequency, daily duration and diversity of purposes of usage.](#_Toc17274)** [5](#_Toc17274)

**[Table S4. The moderating effect of socioeconomic status between daily duration of short-video platforms usage and self-rated health, and relative health deprivation.](#_Toc13644)** [5](#_Toc13644)

**[Table S5. Results for quantile regression analysis of the association between the use of short-video platforms and self-rated health, and relative health deprivation based on propensity score matching with replacement.](#_Toc15157)** [6](#_Toc15157)

**[Table S6. Association between the usage behaviors of short-video platforms and self-rated health, and relative health deprivation with usage behaviors treated continuous variables.](#_Toc5263)** [7](#_Toc5263)

**[Table S7. Association between the usage behaviors of short-video platforms and self-rated health, and relative health deprivation among non-users of short-video platforms.](#_Toc4011)** [8](#_Toc4011)

**[Table S8. The moderating effect of urban-rural residence between daily duration of short-video platforms usage and self-rated health, and relative health deprivation.](#_Toc28554)** [9](#_Toc28554)

# Figures

## Figure S1. The data distribution of self-rated health.


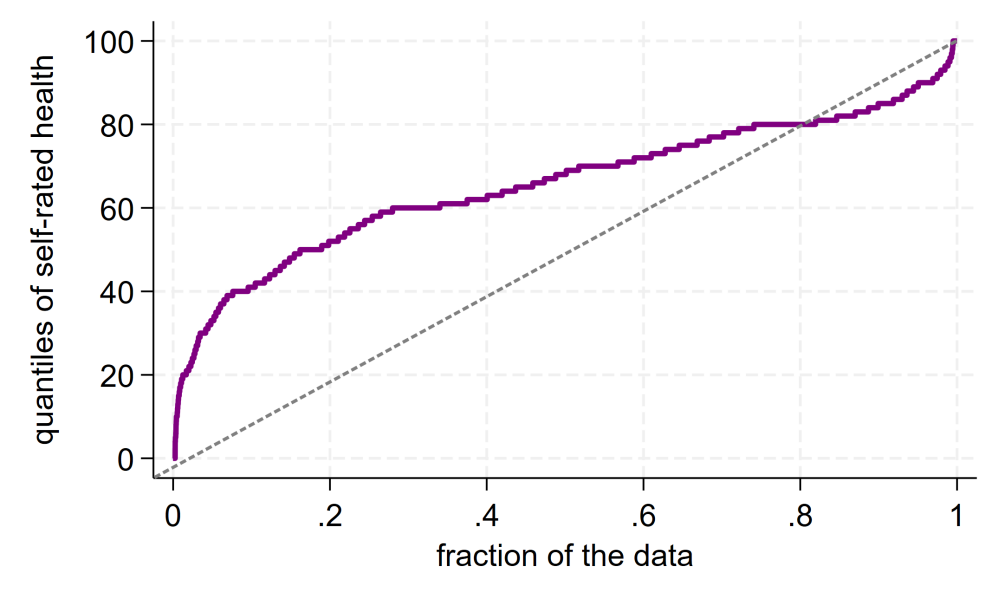


The purple curve fluctuate to a large extent near the grey uniform line, indicating that the scores of self-rated health is not normally distributed.

## Figure S2. The data distribution of relative health deprivation.


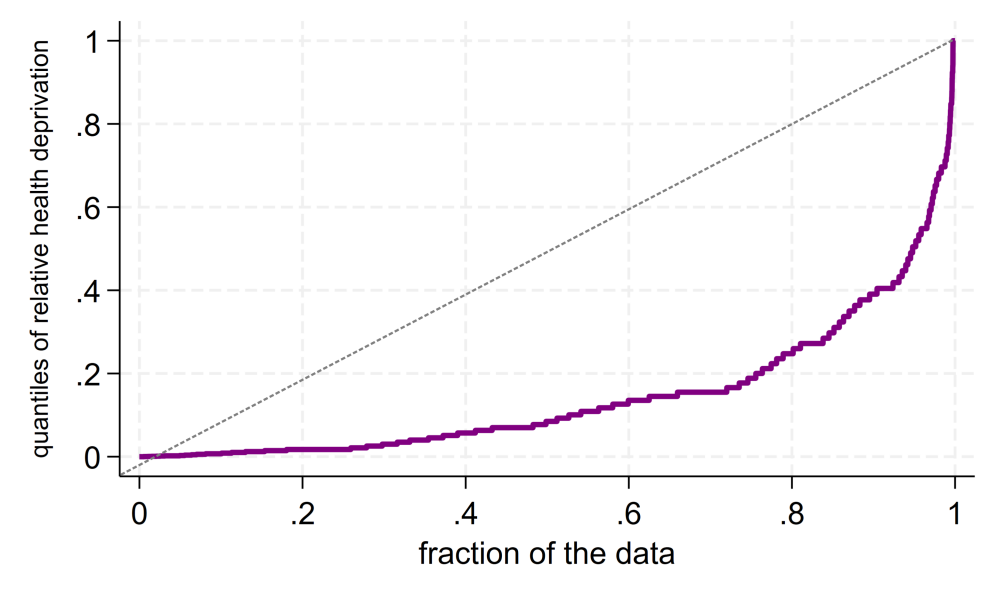


The data distribution of self-rated health. The purple curve fluctuate to a large extent near the grey uniform line, indicating that the scores of relative health deprivation is not normally distributed.

# Tables

## Table S1. Characteristics of the participants in propensity-matched dataset.

| Characteristics^a^ | Short-video platforms | | Self-rated health | | Relative health deprivation | |
| --- | --- | --- | --- | --- | --- | --- |
|  | Users | Non-users | mean(SD) | *P* value | mean(SD) | *P* value |
| **N** | 3,322 | 3,322 |  |  |  |  |
| **Age** | 64.79 (7.27) | 70.21 (8,12) | 64.86 (17.02) | <0.001 | 0.15 (0.17) | <0.001 |
| **Gender** |  |  |  | 0.343 |  | 0.582 |
| Male | 1,558 (46.90) | 1,358 (40.88) | 65.09 (17.18) |  | 0.15 (0.17) |  |
| Female | 1,764 (53.10) | 1,964 (59.12) | 64.69 (16.89) |  | 0.15 (0.17) |  |
| **Marriage** |  |  |  | <0.001 |  | <0.001 |
| Non-spousal | 393 (11.83) | 796 (23.96) | 62.40 (17.84) |  | 0.17 (0.19) |  |
| Spousal | 2,929 (88.17) | 2,526 (76.04) | 65.40 (16.79) |  | 0.14 (0.17) |  |
| **Urban - rural residence** |  |  |  | <0.001 |  | <0.001 |
| Urban | 1,021 (30.73) | 1,010 (30.40) | 67.22 (15.93) |  | 0.13 (0.15) |  |
| Rural | 2,301 (69.27) | 2,312 (69.60) | 63.83 (17.38) |  | 0.16 (0.17) |  |
| **Socioeconomic status** |  |  |  | <0.001 |  | <0.001 |
| Low | 1,266 (38.11) | 1,258 (37.87) | 60.48 (18.12) |  | 0.19 (0.20) |  |
| Middle | 1,805 (54.33) | 1,894 (57.01) | 67.29 (15.69) |  | 0.13 (0.14) |  |
| High | 251 (7.56) | 170 (5.12) | 69.89 (15.81) |  | 0.11 (0.14) |  |
| **Education** |  |  |  | 0.006 |  | <0.001 |
| illiterate / semi-literate | 1,086 (32.69) | 1,856 (55.87) | 63.41 (16.84) |  | 0.16 (0.17) |  |
| Primary school | 1,158 (34.86) | 941 (28.33) | 65.13 (17.57) |  | 0.15 (0.17) |  |
| Middle school | 775 (23.33) | 338 (10.17) | 66.69 (16.44) |  | 0.13 (0.16) |  |
| High school / Vocational school | 264 (7.95) | 151 (4.55) | 68.22 (16.46) |  | 0.12 (0.16) |  |
| College degree / Bachelor's degree or above | 39 (1.17) | 36 (1.08) | 68.95 (13.64) |  | 0.10 (0.14) |  |
| **Employment status** |  |  |  | <0.001 |  | <0.001 |
| Unemployed | 2,031 (61.14) | 2,202 (66.29) | 63.94 (17.42) |  | 0.16 (0.18) |  |
| Employed | 1,291 (38.86) | 1,120 (33.71) | 66.48 (16.17) |  | 0.13 (0.15) |  |
| **The convenience of obtaining medical services** |  |  |  | <0.001 |  | <0.001 |
| Low | 14 (0.42) | 7 (0.21) | 53.52 (26.67) |  | 0.29 (0.32) |  |
| Medium-low | 100 (3.01) | 180 (5.42) | 55.07 (19.09) |  | 0.25 (0.21) |  |
| Middle | 384 (10.48) | 522 (15.71) | 59.08 (18.13) |  | 0.21 (0.20) |  |
| Medium-high | 1,942 (58.46) | 1,946 (58.58) | 66.03 (16.38) |  | 0.14 (0.16) |  |
| High | 918 (27.63) | 667 (20.08) | 67.07 (16.06) |  | 0.13 (0.15) |  |
| **Number of persons living together** |  |  |  | 0.003 |  | <0.001 |
| 0 | 271 (8.16) | 421 (12.67) | 63.66 (16.89) |  | 0.16 (0.17) |  |
| 1-3 | 1,721 (51.81) | 1,630 (49.07) | 64.29 (17.38) |  | 0.16 (0.17) |  |
| 4-6 | 1,161 (34.95) | 1,114 (33.53) | 65.74 (16.74) |  | 0.14 (0.16) |  |
| >6 | 169 (5.09) | 157 (4.73) | 67.24 (14.96) |  | 0.12 (014) |  |
| **Number of persons contacting with regularly** |  |  |  | <0.001 |  | <0.001 |
| 0 | 133 (4.00) | 156 (4.70) | 58.20 (19.55) |  | 0.22 (0.21) |  |
| 1 | 516 (15.53) | 765 (23.03) | 64.60 (17.29) |  | 0.15 (0.18) |  |
| 2-3 | 1,278 (38.47) | 1,255 (37.78) | 65.14 (16.52) |  | 0.15 (0.16) |  |
| 4-5 | 682 (20.53) | 652 (19.63) | 65.69 (15.82) |  | 0.14 (0.16) |  |
| ≥6 | 713 (21.46) | 494 (14.87) | 65.26 (18.06) |  | 0.15 (0.18) |  |
| **Multimorbidity - weighted index** | 2.50 (2.03) | 2.66 (2.09) | 64.86 (17.02) |  | 0.15 (0.17) |  |
| **Depression** | 5.71 (4.64) | 6.65 (5.02) | 64.86 (17.02) |  | 0.15 (0.17) |  |
| **Anxiety** | 1.99 (3.35) | 2.39 (3.71) | 64.86 (17.02) |  | 0.15 (0.17) |  |
| **Loneliness** | 3.63 (1.27) | 3.90 (1.39) | 64.86 (17.02) |  | 0.15 (0.17) |  |

^a^Data are presented as the mean (SD) for continuous variables and number (%) for categorical variables.

## Table S2. Correlation analysis of the usage behaviors of short-video platforms.

| Variables | Years of use | Frequency of use | Daily duration of use | Numbers of purposes of use |
| --- | --- | --- | --- | --- |
| Years of use | 1 |  |  |  |
| Frequency of use | 0.810^a^ | 1 |  |  |
| Daily duration of use | 0.640^a^ | 0.692^a^ | 1 |  |
| Numbers of purposes of use | 0.705^a^ | 0.783^a^ | 0.643^a^ | 1 |

^a^p < 0.001.

## Table S3. Test for multicollinearity of the years, frequency, daily duration and diversity of purposes of usage.

| Variables | VIF | Judgment^a^ |
| --- | --- | --- |
| Years of use | 3.09 | <5 |
| Frequency of use | 4.20 | <5 |
| Daily duration of use | 2.07 | <5 |
| Numbers of purposes of use | 2.80 | <5 |
| Mean VIF | 3.04 | |

^a^VIF<5 indicates that there is no collinearity with other variables and it can be directly included in the model.

## Table S4. The moderating effect of socioeconomic status between daily duration of short-video platforms usage and self-rated health, and relative health deprivation.

|  | N | Self-rated health | | Relative health deprivation | |
| --- | --- | --- | --- | --- | --- |
|  |  | Coefficient (95%CI) | *P* for interaction | Coefficient (95%CI) | *P* for interaction |
| High (ref)^a^ | 535 | — | — | — | — |
| Middle | 4310 | -2.257 (-3.540, -0.975) | 0.001 | 0.018 (0.005, 0.031) | 0.005 |
| Low | 2880 | -2.022 (-3.349, -0.695) | 0.003 | 0.014 (0.001, 0.027) | 0.037 |

^a^Taking the high socioeconomic status as the reference group.

## Table S5. Results for quantile regression analysis of the association between the use of short-video platforms and self-rated health, and relative health deprivation based on propensity score matching with replacement.

| Quantile | Self-rated health | | Relative health deprivation | |
| --- | --- | --- | --- | --- |
|  | Coefficient (95%CI) | *P* value | Coefficient (95%CI) | *P* value |
| Ref: non-users^a^ |  |  |  |  |
| 0.1 | -2.841 (-4.658, -1.023) | 0.002 | -0.003 (-0.005, -0.001) | 0.014 |
| 0.2 | -0.332 (-1.521, 0.857) | 0.585 | -0.004 (-0.008, -0.000) | 0.037 |
| 0.3 | 0.977 (-0.543, 2.497) | 0.208 | -0.005 (-0.009, -0.01) | 0.012 |
| 0.4 | 0.631 (-0.635, 1.897) | 0.328 | -0.002 (-0.006, 0.002) | 0.426 |
| 0.5 | 0.926 (-0.555, 2.408) | 0.220 | -0.003 (-0.010, 0.003) | 0.320 |
| 0.6 | 0.333 (-0.502, 1.168) | 0.434 | -0.005 (-0.015, 0.006) | 0.378 |
| 0.7 | 1.039 (0.016, 2.062) | 0.046 | -0.011 (-0.024, 0.003) | 0.112 |
| 0.8 | 1.339 (0.238, 2.440) | 0.017 | 0.008 (-0.011, 0.028) | 0.401 |
| 0.9 | 2.068 (0.992, 3.143) | <0.001 | 0.038 (-0.003, 0.080) | 0.071 |
| Model |  | |  | |
| Adjusted R-squared | 0.179 | | 0.170 | |
| F-value | 97.67 | | 91.78 | |
| F significance | <0.001 | | <0.001 | |

^a^The sample size that was successfully matched was 5857. Among them, there were 4,403 users and 1,454 non-users.

## Table S6. Association between the usage behaviors of short-video platforms and self-rated health, and relative health deprivation with usage behaviors treated continuous variables.

|  | Self-rated health | | Relative health deprivation | |
| --- | --- | --- | --- | --- |
|  | Model 1,  coefficient (95%CI) | Model 2,  coefficient (95%CI) | Model 3,  coefficient (95%CI) | Model 4,  coefficient (95%CI) |
| Years of use | 2.277 (1.466, 3.089)^a^ | 0.891 (0.136, 1.646)^c^ | -0.020 (-0.028, -0.012)^a^ | -0.008 (-0.016, -0.001)^c^ |
| Frequency of use | -0.124 (-0.693, 0.445) | -0.239 (-0.763, 0.284) | 0.003 (-0.003, 0.008) | 0.004 (-0.002, 0.009) |
| Daily duration of use | -1.049 (-1.598, -0.500)^a^ | -1.051 (-1.555, -0.547)^a^ | 0.012 (0.006, 0.017)^a^ | 0.012 (-0.007, 0.017)^a^ |
| Numbers of purposes of use | 1.523 (0.824, 2.222)^a^ | 0.850 (0.200, 1.499)^c^ | -0.016 (-0.022, -0.009)^a^ | -0.010 (-0.017, -0.004)^b^ |
| Control variables | No | Yes | No | Yes |
| Adjusted R-squared | 0.018 | 0.177 | 0.014 | 0.163 |
| F-value | 37.02 | 93.41 | 27.40 | 84.79 |
| F significance | <0.001 | <0.001 | <0.001 | <0.001 |

^a^p < 0.001; ^b^p < 0.01; ^c^p < 0.05.

## Table S7. Association between the usage behaviors of short-video platforms and self-rated health, and relative health deprivation among non-users of short-video platforms.

|  | Self-rated health | | Relative health deprivation | |
| --- | --- | --- | --- | --- |
|  | Model 1,  coefficient (95%CI) | Model 2,  coefficient (95%CI) | Model 3,  coefficient (95%CI) | Model 4,  coefficient (95%CI) |
| **Years of use** |  |  |  |  |
| ≤1 year (ref) |  |  |  |  |
| 1-3 years | 1.939 (-0.034, 3.912) | 1.287 (-0.547, 3.121) | -0.016 (-0.035, 0.003) | -0.011 (-0.028, 0.007) |
| ≥3 year | 4.286 (2.302, 6.270)^a^ | 2.304 (0.440, 4.168)^c^ | -0.038 (-0.056, -0.019)^a^ | -0.023 (-0.041, -0.005)^c^ |
| **Frequency of use** |  |  |  |  |
| several times a year (ref) |  |  |  |  |
| several times a month | -5.001 (-11.684, 1.681) | -5.818 (-12.013, 0.378) | 0.052 (-0.011, 0.116) | 0.060 (0.000, 0.120) |
| 1-4 times a week | -4.333 (-10.429, 1.763) | -5.022 (-10.675, 0.630) | 0.039 (-0.019, 0.097) | 0.045 (-0.010, 0.099) |
| almost every day | -5.035 (-11.059, 0.988) | -5.494 (-11.074, 0.085) | 0.050 (-0.007, 0.107) | 0.053 (-0.001, 0.107) |
| **Daily duration of use** |  |  |  |  |
| ≤30 minutes (ref) |  |  |  |  |
| 31-60 minutes | 1.087 (-0.343, 2.518) | 0.015 (-1.320, 1.349) | -0.008 (-0.022, 0.006) | -0.001 (-0.014, 0.012) |
| 61-120 minutes | -2.449 (-4.097, -0.801)^b^ | -3.38 (-4.923, -1.854)^a^ | 0.027 (0.012, 0.043)^b^ | 0.32 (0.018, 0.047)^a^ |
| 120-240 minutes | -2.718 (-4.953, -0.482)^c^ | -2.732 (-4.804, -0.661)^c^ | 0.032 (0.011, 0.053)^b^ | 0.033 (0.013, 0.053)^b^ |
| ≥240 minutes | -0.127 (-3.163, 2.908) | -0.297 (-3.111, 2.518) | 0.008 (-0.021, 0.037) | 0.012 (-0.015, 0.039) |
| **Numbers of purposes of use** |  |  |  |  |
| 1 (ref) |  |  |  |  |
| 2 | 1.660 (0.413, 2.907)^b^ | 1.265 (0.102, 2.428)^c^ | -0.016 (-0.028, -0.004)^b^ | -0.011 (-0.022, 0.000) |
| ≥3 | 2.716 (1.113, 4.320)^b^ | 1.555 (0.048, 3.061)^c^ | -0.029 (-0.044, -0.014)^a^ | -0.020 (-0.035, -0.006)^b^ |
| Control variables | No | Yes | No | Yes |
| Adjusted R-squared | 0.014 | 0.157 | 0.015 | 0.132 |
| F-value | 6.65 | 33.88 | 7.10 | 27.66 |
| F significance | <0.001 | <0.001 | <0.001 | <0.001 |

^a^p < 0.001; ^b^p < 0.01; ^c^p < 0.05.

## Table S8. The moderating effect of urban-rural residence between daily duration of short-video platforms usage and self-rated health, and relative health deprivation.

|  | N | Self-rated health | | Relative health deprivation | |
| --- | --- | --- | --- | --- | --- |
|  |  | Coefficient (95%CI) | *P* for interaction | Coefficient (95%CI) | *P* for interaction |
| Urban (ref)^a^ | 2342 | — | — | — | — |
| Rural | 5383 | -0.890 (-1.667, -0.113) | 0.025 | 0.008 (0.000, 0.016) | 0.038 |

^a^Taking the urban residence as the reference group.
